# Infant and Early Childhood Mental Health (IECMH) and Early Childhood Intervention: Intentional Integration

**DOI:** 10.3390/ijerph21070870

**Published:** 2024-07-03

**Authors:** Neal M. Horen, Julia Sayles, Kelli McDermott, Kirsten Sippel-Klug, Jennifer Drake-Croft, Toby Long

**Affiliations:** Center for Child and Human Development, Georgetown University, Washington, DC 20007, USA; jks116@georgetown.edu (J.S.); km1681@georgetown.edu (K.M.); kirstensippelklug@yahoo.com (K.S.-K.); jad421@georgetown.edu (J.D.-C.); longt@georgetown.edu (T.L.)

**Keywords:** infant and early childhood mental health, early intervention, system of care, child development, early relational health, early childhood policy, pyramid model, interdisciplinary

## Abstract

Infant and Early Childhood Mental Health (IECMH) is a multidisciplinary field of inquiry, practice, and policy concerned with enhancing the social-emotional competence of infants and young children. Early Childhood Intervention (ECI) is a system of services that supports infants and toddlers with disabilities and their families. ECI providers promote a child’s development in all domains, including social-emotional. The purpose of this paper is to describe how two systems, Early Childhood Intervention and Infant Early Childhood Mental Health, collaborate when serving children who have developmental delays or disabilities and their families and other caregivers. We will discuss two models used to promote social-emotional development, the Pyramid Model and IECMH Consultation, and provide three examples that demonstrate how ECI and IECMH intersect at both the family, classroom, and system levels.

## 1. Introduction

Infancy and early childhood represent a period of rapid brain, body, and skill development that provides the foundation for future learning, behavior, and health [1]. The science of early child development is understood as a series of complicated relationships among fields such as neuroscience, developmental psychology, biology, and genomics [2].

Brain development is influenced by early life experiences [3]. From birth, our brains grow and adjust to our physical environment, interpersonal interactions, and evolving skill development [4]. These repetitive, consistent, predictable, and nurturing experiences are the “active ingredients” required to express the underlying genetic potential of each child and occur in the context of relationships [5,6].

This early period during which the brain is exquisitely sensitive to the positive aspects of caregiving also renders it vulnerable to stressors. Infants who are exposed to early life stressors (ELS), such as severe adversity (domestic violence and caregiver substance use), and severe relational poverty (caregiver neglect and lack of caregiver attunement), or biological insults, such as prematurity or disability, may experience long-term developmental and health consequences [4]. Promoting positive experiences for all young children is crucial to mitigating the impacts of early life stressors.

Molecular biology, genomics and epigenetics, neuroscience, and social science inform us that there is a clear and direct relationship between various external factors and brain development [7]. Economic research has affirmed the advantages of a positive, high-quality early childhood education for individuals, especially those who are most disadvantaged, in terms of long-term health, education, employment, and social outcomes [8]. Family research indicates that consistent family routines support education success [9]. Thus, it is critical for early childhood health, education, and social service providers to be aware of those strategies that are most likely to support young children, their families, and communities.

The changing global landscape and its impact on children only increase the need for strategies that support young children. The number of children who are considered vulnerable is growing globally due to a variety of factors including conflicts, climate change, socio-cultural adversity, and disability. Globally there are 250 million children under the age of five who are at risk for not reaching their developmental potential [10]. Over 400,000 infants and toddlers with disabilities or developmental delays receive services in the United States under a comprehensive federally mandated Early Childhood Intervention program (ECI also referred to as EI).

ECI is defined as a “social model” composed of multisectoral and transdisciplinary services that are based on the Convention on the Rights of the Child (CRC) and the Convention on the Rights of Persons with Disabilities (CRPDs). ECI service providers include public institutions and civil society organizations that offer family-centered, coordinated, intensive, and individualized services to improve child development and resilience and strengthen family competencies, communications, and skills to improve child development. ECI services often include advocacy for the educational and social inclusion of children and their families, and they work to prevent and mitigate child abuse, neglect, and abandonment. Contemporary ECI service providers also assist with the deinstitutionalization of children from institutions, support family placement services, and the families receiving children. Many children receiving ECI could benefit from the support of IECMH providers due to difficulty with regulation, behavioral challenges, and social skill development for example.

Like ECI, the provision of IECMH support encompasses a range of multisectoral and transdisciplinary clinical services to recognize and address the social-emotional needs of babies, young children, and their families [11]. Although estimates indicate that one in six U.S. children aged 2–8 years (17.4%) had a diagnosed mental, behavioral, or developmental disorder there are no accurate estimates of the number of children receiving IECMH services or supports [12]. However, in the United States, the 16,217 Head Start programs, the federally funded early care and education programs for disadvantaged toddlers and preschoolers, are supported by IECMH consultants [13]. Brown, Copeland, Sucharew & Kahn [14] found that 24% of 3 and 4-year-olds in low-income clinical settings screened positive for social-emotional problems. Many children who receive IECMH support also experience developmental delays or differences and could benefit from ECI services [15].

Many professionals working with young children, both with and without disabilities or developmental delays, often assume that mental health is not within their scope of practice [16,17]. This assumption can be a missed opportunity, as studies indicate that children with developmental delays (DDs) or disabilities experience more emotional and behavioral difficulties [18], and children experiencing social challenges often show delays, especially in the language area [19].

According to Thurm et al. [20], children with language delays exhibit significantly higher levels of social-emotional and behavioral issues compared to their typically developing peers at 18 and 24 months of age. Moreover, the severity of the language delay is directly commensurate with the intensity of social-emotional and behavioral problems [20]. This relationship between language delays and emotional/behavioral issues has also been observed in a study examining the effectiveness of language intervention in reducing behavior problems in young children with language delays [18]. Additionally, Cheng [21] discovered a similar association between cognitive delays and behavior problems. Children with cognitive delays displayed more behavior problems than their peers at 9 and 24 months of age. By the age of 5, these behavior problems persisted.

Professionals who serve families of children with disabilities, such as pediatricians, speech therapists, occupational therapists, and physical therapists, acknowledged that children with disabilities or developmental delays and their caregivers may benefit from mental health support, however, they are unsure of their role and responsibilities or where to refer caregivers for services or support [16].

The field of IECMH continues to emerge and develop. As it is a cross-disciplinary workforce, there is currently no clear pathway to becoming a specialized practitioner in this area. This often results in clinicians embracing the value of IECMH, but lacking the training and expertise to practice competently [11]. Additionally, the workforce shortage in both the mental health and early childhood fields compounds challenges to the availability of well-trained IECMH providers. Among the many efforts to develop a well-qualified IECMH workforce are the Center of Excellence for Infant and Early Childhood Mental Health Consultation www.iecmhc.org (accessed on 28 April 2024), The Alliance for the Advancement of Infant Mental Health www.allianceaimh.org (accessed on 28th April 2024), and the Pyramid Model www.challengingbehavior.org (accessed on 28th April 2024). None of these pathways emphasize a comprehensive understanding of early childhood interventions focused on developmental needs outside of social and emotional development.

Developing collaborative relationships between ECI and infant and early childhood mental health practitioners has the potential to facilitate and improve appropriate support for all families by ensuring that all providers working with families have a nuanced understanding of development. Unfortunately, the ECI and the IECMH systems are often created under different administrative structures. ECI is typically part of the education or healthcare systems while IECMH is often part of behavioral healthcare, child protection, or mental health and substance use services [15]. A well-established early childhood system supports integrated service delivery mechanisms building on the strengths of each system to foster a child’s development, identify potential needs early on, and intervene appropriately. An integrated early childhood system requires collaborative partnerships among behavioral healthcare, early childhood intervention, and other child-serving systems such as child protection and early education.

The purpose of this paper is to describe the importance of collaboration between early childhood intervention (ECI) teams and infant early childhood mental health (IECMH) practitioners in promoting a child’s developmental and functional skill acquisition, engagement, and social relations [22]. We will discuss the intersection of ECI and IECMH, describe three evidence-based practices used to provide comprehensive support and provide three examples of how ECI and IECMH intersect at the family, classroom, and system levels.

## 2. Infant and Early Childhood Mental Health

Infant and Early Childhood Mental Health (IECMH) is a system that offers a range of supports and services on four levels of intensity: promoting healthy social-emotional development for all young children, preventing challenging behaviors through parenting support and home visiting programs, identifying concerns, delays, and behavioral challenges through screening and evaluation, intervening to address family risk factors such as abuse and neglect, substance use disorder, domestic violence, and parental mental health issues, and treating to address parent–child relationships when there is a clinically significant impact on the child and their family. IECMH includes a variety of direct and indirect services to young children, their families, and other caregivers. Providers include psychologists, social workers, and specially trained early childhood mental health consultants. Although a relatively new field, research indicates that providing support to children and families early mitigates the negative effects of adversity, promotes resiliency), and promotes positive behavior [23].

## 3. Early Childhood Intervention

Early Childhood Intervention is a component of the US federal law, Individuals with Disabilities Education Act [24], that defines the services provided to infants and toddlers identified with disabilities or developmental delays aged birth to 3 or 4 years of age. ECI includes a variety of therapeutic, educational, development, and health-related services that are individualized to meet the family’s prioritized concerns. Examples of these services include, but are not limited to, early childhood special education, physical therapy, occupational therapy, speech therapy, and parent training. The law stipulates that while ECI is not the sole agency responsible for offering these services, it is the agency mandated to facilitate collaboration among local resources and agencies. From the outset, the necessity of collaboration was recognized and incorporated into the law.

Determining eligibility for this program is performed by assessing the five fundamental domains of development: physical (fine and gross motor), social-emotional, cognitive, self-help, and communication (receptive and expressive). The primary goals of ECI are to build the capacity of parents and caregivers, and to foster the child’s development through daily activities and routines, enhancing their quality of life [25,26,27].

Early Childhood Intervention programs have established outcome measures to capture a child’s growth and participation in meaningful family and community routines. These outcomes reflect the integrated nature of child development [15]:

1. Child has positive social-emotional skills (e.g., social relationships);
2. Child acquires and uses knowledge and skills (e.g., early language and communication);
3. Child uses appropriate behaviors to meet their needs.

## 4. The Intersection of ECI and IECMH

Promoting a child’s social-emotional development is one of the identified outcomes of ECI and is the purpose of IECMH. Social-emotional development is the emerging ability of young children (ages birth-5) to “form close and secure adult and peer relationships; experience, regulate, and express emotions in socially and culturally appropriate ways; and explore the environment and learn all in the context of family, community, and culture” [28]. Foundational social-emotional skills include, but are not limited to, the expression and management of a wide range of emotions, perspective-taking, empathy, inhibitory control, self-confidence, and friendship skills [29,30,31,32]. Despite the growing interest in this area over the past decade [33], ECI providers pay less attention to social-emotional development compared to physical, communication, or cognitive development [17].

Smith and his colleagues hypothesized that the decreased emphasis on social-emotional development, compared to other areas, was likely a result of ECI providers lacking competence and confidence when addressing issues related to IECMH. Dickinson found that ECI professionals (1) lacked a systematic approach to determining suitable interventions for challenging behaviors, (2) were not adequately trained in implementing functional behavioral assessment and interventions, and (3) were unaware that their training did not fully prepare them to work with young children exhibiting challenging behaviors [34]. On the other hand, children who are displaying challenging or other behaviors that indicate a need for social-emotional support are often referred to IECMH services. While these services likely focus on promoting a positive classroom environment and developing social-emotional competencies and skills, there is an indication that many young children displaying such classroom behavior are not identified as having developmental delays, especially in the language area [19]. A more robust knowledge of early childhood developmental milestones and a broad developmental screening process, rather than the narrow focus of only social-emotional screening, within IECMH, could facilitate more referrals to ECI for formal evaluation [11].

**Figure 1:**
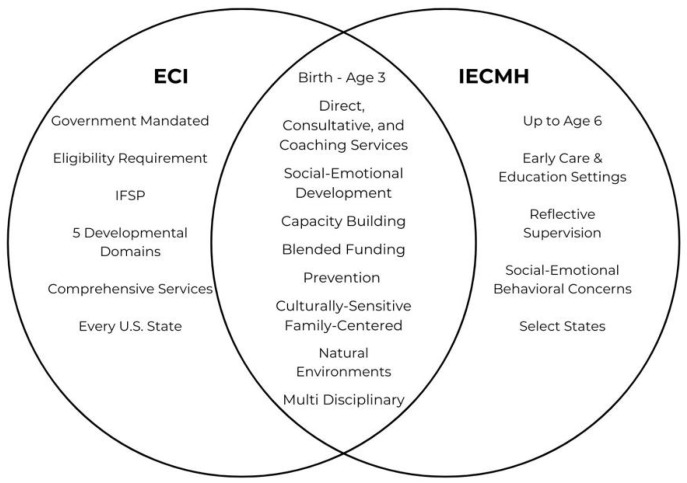
Intersection Between Early Intervention and IECMH.

The importance of promoting a child’s development, nurturing parent–child relationships, and supporting the social-emotional and mental health needs of infants and toddlers is recognized by both the early intervention and behavioral healthcare systems (Figure 1), however, there is an indication that ECI providers are less confident in their capacity to employ strategies that enhance the parent–child relationship and the social-emotional capacities of the child [16]. Recently, United States-based technical assistance centers such as the Early Childhood Technical Assistance Center [15], the National Center for Pyramid Model Innovations https://challengingbehavior.org/ (accessed on 28 April 2024), and the Center of Excellence on Infant and Early Childhood Mental Health Consultation https://www.iecmhc.org/ (accessed on 28 April 2024), state ECI programs, and universities have produced a variety of products to build providers’ capacity to promote healthy social-emotional development, address challenging behaviors, and promote participation in everyday activities and routines.

## 5. Early Childhood System of Care

Comprehensive early childhood development is best realized when infants and young children have access to systems that effectively recognize and meet their developmental needs. Georgetown University Center for Child and Human Development’s conceptual model of early childhood service is based on the system of care (SoC) approach [35], informed by years of providing technical assistance on growing and sustaining early childhood systems. The services and supports of an early childhood system are based on the infrastructure, evaluation process, funding, and workforce development available to ensure an adequate number of competent practitioners to meet child, family, and community needs who collaborate to ensure comprehensive services across child-serving systems (physical health, behavioral health, early intervention, education, and social services).

Implicit in this model is the concept of relational health which posits that “healthy and positive child development emerges best in the context of nurturing, warm, and responsive early parent/caregiver–child relationships when children are surrounded by safe communities with strong trust and social connectedness” [36]. The System of Care model indicates that the health and wellness of each child depends on the health of the entire ecosystem, not just on one individual or family. A continuum of early childhood support and services is needed to (1) promote development by strengthening adult and child interactions, (2) identify developmental challenges early, (3) prevent and reduce factors that can disrupt development, (4) intervene to promote skill development and (5) serve children and families through evidence-based, evidence-informed, or promising interventions to develop responsive and nurturing relationships [37,38,39,40].

Five core strategies have been identified as essential to make the systemic changes necessary for addressing the early childhood system: (1) implementing policy and partnership changes; (2) developing or expanding an array of services and supports based on the SoC approach; (3) creating or improving financing strategies; (4) implementing workforce development and training strategies; and (5) generating support among key stakeholders through strategic communications [35]. Although these strategies are well accepted in systems development, collaboration continues to be challenging, policy change is slow, financing is difficult, and sustainability is nettlesome. Two evidence-based approaches, The Pyramid Model [41] and IECMH Consultation [23], have been shown to be easily integrated into both early intervention and infant and early childhood mental health components of the system.

## 6. Pyramid Model: A Cross-Disciplinary Approach

**Figure 2:**
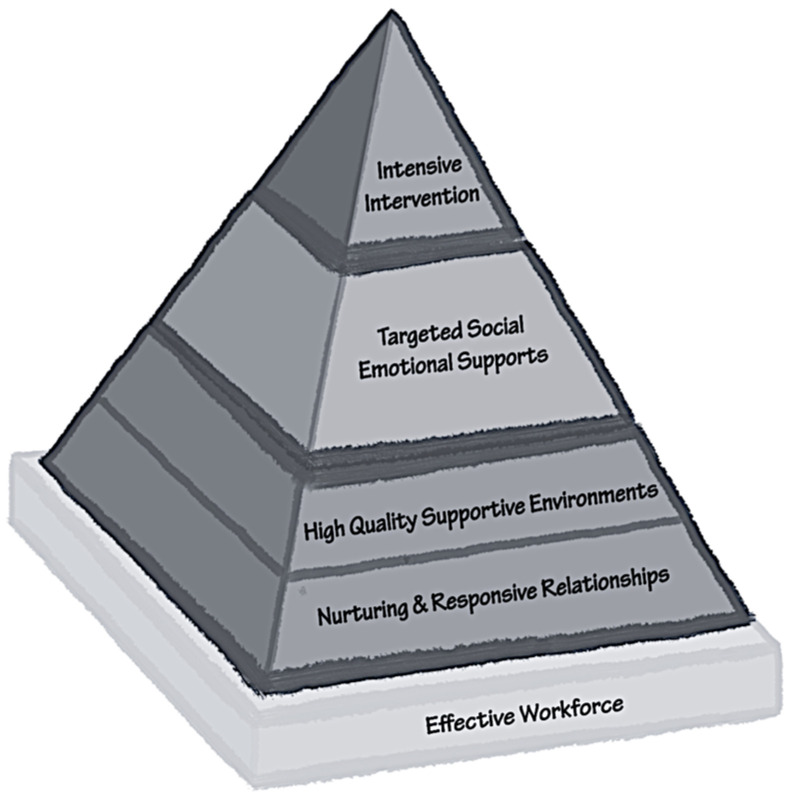
The Pyramid Model (Used with permission from the National Center for Pyramid Model Interventions, 2023).

The Pyramid Model is an evidence-based practice for promoting social-emotional competence in infants and young children. This equity-focused, multi-tiered system of support can be used across a variety of settings that serve children from birth to age 5 [41]. The Pyramid Model builds the capacity of adults to support a child’s social-emotional development through nurturing and responsive relationships, creating high-quality supportive environments, offering developmentally appropriate social-emotional teaching strategies, and creating individualized interventions for children as they are needed [42]. This multi-tiered framework recognizes that at its foundation, a system is built upon an effective workforce that includes well-trained, competent, confident infant and early childhood personnel: mental/early relational health professionals, early interventionists, early care and education staff, preschool teachers, and caregivers. The model supports this workforce through training, technical assistance, and reflective supervision [38] (see Figure 2).

The Pyramid Model practices promote brain development, support regulation, differentiate the individualized cues and communication styles of children, and recognize variations in temperament to foster social-emotional learning and development. Knowledge of social-emotional developmental milestones, the relationship between social-emotional development and behavior, and their relationship to other developmental domains (physical, cognitive, and communication) is key to implementing the practices competently. The model recognizes the importance of safe and secure early relationships, attachment, and cultural responsiveness; thus, an emphasis is placed on building relationships with families and supporting the caregiver–child relationship. The Pyramid Model recognizes that from birth, infants and babies have the capacity to form relationships, express feelings and emotions, lay the foundation for self-regulation through early relationships with caregivers, explore their environments, and begin to develop emotional literacy through nurturing and responsive relationships and high-quality environments. Given that early intervention is grounded in a family-centered, developmental, and equitable approach to service delivery, the Pyramid Model is a natural fit for ECI.

Also aligned with the principles of ECI, the Pyramid Model practices consider the diverse cultural and linguistic backgrounds of infants, young children, and families and how this may inform behavior and social-emotional development. Pyramid practices support cultural awareness and responsiveness through the use of a variety of culturally responsive tools, materials, and strategies. Aligned with trauma-informed care principles, the Pyramid Model supports early childhood personnel to meet the needs of young children and families impacted by trauma [43].

Infants and toddlers with or at risk for disabilities may also have attachment challenges and difficulty learning to regulate their emotions and behavior [44]. There is a relationship between communication delays or disorders and challenging behaviors [17]. Since its inception, the Pyramid Model practices have been designed to promote and support the social-emotional competence of all children, including those identified as having or being at risk for developmental disabilities [45]. The model is divided into increasingly intense tiers of support.

### 6.1. Tier 1: Universal Promotion Practices

Tier 1 of the pyramid represents the universal practices of responsive and nurturing relationships and high-quality supportive environments that benefit the social-emotional development of all children. These practices lay the groundwork for the emotional competencies needed to thrive in relationships by emphasizing the importance of consistency and repetition by the adults who care for them.

Tier 1 practices focus on those daily caregiving routines and activities that create important foundational brain connections and functional skills such as holding, rocking, bathing, feeding, dressing, and talking to infants. Early childhood intervention is routines-based; thus, ECI practitioners are uniquely qualified to incorporate nurturing, relational practices into their support for skill development [46].

### 6.2. Tier 2: Targeted Social-Emotional Supports

Tier 2 of the pyramid highlights targeted social and emotional support. This tier shifts from the universal promotion of social-emotional development to strategies that support skill building and prevention of challenging behavior. Social-emotional skills support the emergence of emotional literacy or the ability to understand one’s own emotional experience and that of others [47]. Emotional literacy supports relationship development with peers, problem-solving, and managing the “big feelings” children experience as they grow (e.g., frustration, worry, excitement, anger, and fear).

Early emotional literacy skills emerge through the repeated experience of responsive relationships and safe environments, which allow a baby to feel safe and secure in expressing emotions [48]. Adults support early emotional literacy by responding to babies with empathy and providing language to their emotional experience (e.g., “You are so happy to see yourself in the mirror. You have such a big smile!”). Early interventionists incorporate Tier 1 and Tier 2 concepts throughout their intervention, especially when coaching a family to recognize their baby’s individual cues, co-regulate, and narrate through everyday activities and routines [46].

### 6.3. Tier 3: Intensive Interventions

The “top of the pyramid” focuses on individualized interventions for children who display behaviors that are impacting their development, learning, and/or relationships. In addition to children with lived experiences (e.g., prematurity, prenatal substance exposure, witnessing interpersonal violence, and attachment disruptions) that could impact social-emotional development, some children have more subtle presentations (e.g., a toddler who has difficulty attending during circle, or a preschooler with language delay struggling to share with peers) that, without support, could impact their early relational health. For example, a baby who cries for most of the day might receive less positive attention and physical closeness than a less fussy baby as excessive crying increases parental stress and disrupts the capacity for parenting [49]. On the other hand, a baby who is quiet and hard to engage might be left alone more frequently because caregivers do not feel connected to them [50]. Both of these children may receive less positive attention and interaction which may delay social development (e.g., responsive smiling). Tier 3 strategies emphasize the crucial role of the family and align with the family’s values and priorities. In addition to implementing Tier 1 and 2 strategies, an early interventionist incorporates specific Tier 3 strategies such as individualized strategies to strengthen an infant’s relationships, emotional expression, exploration of their environment, and emerging play skills.

## 7. Early Childhood Mental Health Consultation: The Georgetown Framework

One service within the field of IECMH is Infant and Early Childhood Mental Health Consultation (IECMHC). IECMHC is an evidence-informed intervention where mental health professionals collaborate with practitioners who work with and care for children from birth to 6 years of age to promote social-emotional development and early relational health [51,52]. This multi-level intervention is implemented by mental health consultants who have training in early childhood development and the practice-based principles of IECMHC: relationship-based care, collaboration, individualization, cultural and linguistic responsiveness, knowledge of development, scientifically-based care delivered in community-based natural settings, services, and supports, and the continuum of strategies (promotion through intervention) [53].

Similar to ECI the primary focus of IECMHC is increasing the capacity of adults caring for young children through consultation and coaching. Mental health consultants form collaborative relationships with programs, teachers, families, and other practitioners working with young children to build their capacity to address or prevent challenging behaviors while simultaneously supporting social-emotional competencies. Consultants join with early childhood professionals to work across natural settings, offering strategies, sharing evidence-based intervention approaches, and facilitating referrals as appropriate [54]. Consultation is strengths-based and considers the contextual and cultural influences on a young child’s behavior. IECMHC is most frequently used in early care and education programs but is becoming more common across other settings including home visiting [55]. The key to an effective IECMHC is competent providers. The Georgetown University Center for Excellence in IECMH Consultation has identified a set of competencies needed by providers to effectively serve young children their families, and caregivers [56].

A growing body of research supports the use of IECMHC to promote positive outcomes for children, families, teachers and staff, and programs. A large multistate program evaluation [57] indicated that IECMHC impacts at various levels including positive outcomes at the child, caregiver, and programmatic level, such as reduced externalizing behavior, reduced job stress and burnout, and increased appropriate referrals to mental health specialists [23].

## 8. ECI and IECMH in Action

Oftentimes IECMH and ECI services exist in isolation. The three ECI outcomes described above indicate the potential for practice alignment and collaboration in service delivery. The following exemplify how ECI providers and IECMH practitioners implement IECMH strategies to promote child development, appropriate behavior, positive parenting skills, classroom management, and system-wide integration. It is instructive to look at this integration at a child and family level, classroom level, and systems level.

### 8.1. Family Level Vignette: The Marks Family

Cecelia Marks, a 15-month-old, lives in a suburban townhouse with her parents, with a cultural background originating from the South Pacific Islands. Mr. Marks, who grew up in the Philippines speaks English and Tagalog with Cecelia while Mrs. Marks, who grew up in Germany, speaks German and Tagalog. Both parents are proud that they are raising their daughter to be multicultural and would like her to be bilingual. Cecelia was referred to a local Early Childhood Intervention (ECI) program by her pediatrician due to concerns about her development and possible signs of autism. At the initial screening, the ECI provider recommended a full evaluation of Cecelia’s development. Cecelia’s parents declined the evaluation at this point because they were skeptical that she had delays in her development or had ASD. The ECI provider gave the family various resources and information, reassuring the family of the opportunity to self-refer in the future.

As Cecelia turned two, Mr. And Mrs. Marks requested an evaluation from the ECI program because of concerns about Ceceli’s behavior. They described Cecelia as having little language (in any of the three languages) and displaying challenging behaviors, including frequent screaming and toy-throwing. These behaviors intensified in the presence of her father, whereas her mother reported having the ability to anticipate and manage her needs more effectively. Because the home was bilingual, the evaluation and subsequent services were provided in German and English.

The ECI team explained that the plan would focus on the family’s concerns, giving them insights and strategies on how to facilitate their daughter’s development. The multidisciplinary assessment, conducted in the family’s home with both parents present indicated significant delays in Cecelia’s communication, social, cognitive, and self-help skills. The evaluation also highlighted Cecelia’s strengths, especially in non-verbal communication and her ability to engage in play when visual cues and a structured environment were provided. The Marks provided a detailed description of Cecelia’s day, expressing their main concern about her behavior, which they described as constant climbing, jumping, and screaming. Mrs. Marks also noted that Cecelia’s play seems more chaotic compared to other children she knows, and she expressed her bewilderment as to why her daughter exhibits such challenging behaviors. Furthermore, Mrs. Marks expressed her exhaustion, as Cecelia consistently goes to bed as late as 11 pm and refuses to sleep in her own bed.

Based on the concerns of the family and Cecelia’s development, the ECI providers and parents decided to focus on building Cecilia’s communication skills, helping Mr. and Mrs. Marks develop positive parenting skills, and promoting co-regulation. A variety of participation-based outcomes were included in the Individualized Family Service Plan and a schedule of weekly home visits was established.

The team determined that they would begin by using strategies to help parents learn about co-regulation and how to help Cecelia participate more positively in her daily routines. The team implemented Pyramid Model strategies to help Cecelia participate more positively in her daily routines, support her positive engagement, and foster her communication development. Using Pyramid Model practices https://challengingbehavior.org/ (accessed on 28 April 2024), the ECI providers helped Mrs. Marks create a visual schedule and taught Mr. and Mrs. Marks how and when to refer to it to reinforce consistency and predictability in Cecelia’s daily routine. They taught them to use positive reinforcement techniques, how to ignore unacceptable behavior, how to have developmentally appropriate expectations, and introduced them to available toddler-friendly community resources such as the library story time. They also taught Mr. and Mrs. Marks how to co-regulate with Cecelia, including deep breathing, calming, and stroking her back or arm. By first addressing her difficulties around emotional regulation, the family was better poised to implement the other strategies.

Cecilia’s journey through ECI was complex. After 6 months of intervention, she continued to struggle with regulation and her language delay was becoming more apparent. Both parents were concerned and frustrated about Cecelia’s lack of progress. The ECI team suggested that the family complete a Behavior Support Planning Document to better understand Cecelia’s behavior, identify desired skills, and develop a plan on how to promote them. Although many positive changes were made creating better parent–child interactions, over the next few months, Mr. And Mrs. Marks continued to have difficulty understanding Cecelia’s behavior and responding appropriately. After an instance of an inappropriate reaction to Cecelia’s behavioral outbursts, the ECI team suggested the family receive support from the IECMH system. The MH consultant suggested that the family may benefit from Parent–Child Interaction Therapy (PCIT), an intensive, specialized mental health support to address the ways their relationship that may be impacting Cecilia’s development and to further develop their skills and increase their confidence in parenting. The ECI therapists and the PCIT therapist collaborated to reinforce each other’s strategies during their sessions and created a comprehensive, integrated plan.

### 8.2. Classroom Vignette: Friends Child Development Center

Jacob Clark is an 18-month-old little boy who lives with his mother, father, and older sister Jasmine. Both Jasmine and Jacob attend the Friends Child Development Center (FCDC) 9 h/day. Jacob’s classroom teacher has been concerned about Jacob’s behavior for a few months and has asked for support from the Infant Early Childhood Mental Health Consultant. The consultant meets with Jacob’s teacher weekly for reflective supervision, providing suggestions on how best to help Jacob regulate his behavior, calm down prior to nap time, and promote language development. After about 3 months Jacob’s behavior escalated rather than progressing as the consultant expected. When queried, the teacher indicated she was also getting more concerned about Jacob’s language development. Jacob has few words, although he has started to make a variety of sounds. The Director of the FCDC referred Jacob and his family to the Early Childhood Intervention Program for a developmental evaluation.

Both parents were present for the evaluation, which took place at the FCDC in a separate room when Jacob was about 2 years old. Both parents mentioned that they were aware of Jacob’s slight developmental delay, and they too were beginning to be concerned about his behavior. He was having more difficulty staying in bed through the night and he had become what they described as a “picky eater”, preferring only soft foods.

The evaluation results indicated that Jacob’s motor skills were average for his age as well as his non-verbal problem-solving. He was showing a significant delay in his language skills and the evaluators also noted that he was showing impulsiveness and a short attention span, even for a two-year-old.

Jacob’s parents have a close relationship with him, describing him as having a feisty spirit. As noted by his teachers, he uses simple vowel sounds although he will say “hi”, “bye”, and “Mum and Dada”. Rather than using gestures to let others know what he wants, he will often just take what he wants, scream, or whine. Jacob engages in social games with his father and enjoys physical play and animated expressions. He is still learning to interact with books as he does not sit through an entire simple, short story. Overall, his family is happy with Jacob but are beginning to be more concerned about his language, behavior, and eating skills.

Because Jacob spends so much time at the FCDC and his behavior is more worrisome there, the ECI services will be provided there on a weekly basis. The team, including the IECMH Consultant, developed a plan that would address Jacob’s language, eating skills, and behavior. Since feeding was a major concern at the FCDC also, the weekly ECI service was scheduled during breakfast time at the FCDC followed by reflective supervision among the IECMH consultant, teacher, and the ECI primary service provider, a speech-language pathologist. The primary service provider also made a home visit twice monthly to support Jacob’s parents with mealtime and bedtime routines. This was also an opportunity for the team to ensure consistency between the FCDC and home.

The IECMH consultant also supported Jacob’s interaction with his teachers and other children. The team also developed a different perspective of the Clark’s. Given the family history of developmental difficulties including autism, the consultant helped the team recognize that the Clark’s may be feeling fearful. Through the consultative relationship, teachers were able to identify needs and actionable ways to strengthen their relationship with the family through a strengths-based stance of curiosity versus judgment.

The primary service provider and the IECMH consultant collaborated to develop a variety of strategies that promoted Jacob’s language development, regulation, and feeding skills. Through the use of signing, Jacob’s use of words started to make progress, he also became less impulsive as the classroom atmosphere became more relaxed and less chaotic.

The IECMH consultant also worked with the program director to address her concerns about having sensitive conversations with families and how to best deliver sensitive information. The consultant and director worked together to identify training topics for the program staff to offer guidance and support on how to have these conversations as well as to identify developmental concerns early and how best to collaborate with ECI professionals in the classroom.

### 8.3. System Level Vignette: North Carolina

The North Carolina Early Intervention (NCEI) is part of the state’s Department of Health and Human Services Division of Child and Family Well-being. Sixteen Children’s Developmental Services Agencies (CDSAs) are located across the state and work with local service providers to support families and their children during the critical birth to three developmental period. North Carolina identified improving social-emotional outcomes for children and families in Early Intervention as a goal and formed a variety of workgroups to make recommendations to support this work. The evidence-based practices workgroup proposed implementing (a) coaching in Natural Learning Environment Practices (NELP) to improve interactions with families, (b) the Pyramid Model to support social-emotional goals, and (c) developing a cross-system infrastructure.

The first phase of implementation included training to ECI providers across the state on coaching in Natural Learning Environments Practices and the Pyramid Model. This training built the foundation for the Pyramid Model train-the-trainer system as this first group of providers trained others at the local level. Over a two-year period, North Carolina trained small cohorts to be local trainers who in turn trained ECI providers who support the social development of young children. As more providers became competent in incorporating Pyramid strategies into NELP, ECI providers and programs explored the benefits of being trained and certified in the use of other interventions such as Circle of Security (CoS) [58], Positive Parenting Program (Triple P) [59], Child–Parent Psychotherapy (CPP) [60], and Attachment and Biobehavioral Catch-up (ABC) [61].

Building the capacity of ECI providers in the Pyramid Model’s framework and tools is the foundation of the Pyramid Model. Training has enhanced ECI providers’ competence and confidence in supporting a young child’s social-emotional skills and collaborating with IECMH consultants. Likewise, the IECMH consultants recognize the intersection of social-emotional development with other developmental domains, especially language development. Based on the training and technical assistance provided, the early childhood system within North Carolina is more cohesive, the providers are more collaborative, and the children and families are better served. Based on feedback from the providers, the consultants, and the trainers, North Carolina is planning to provide a system of reflective supervision for all practitioners as well as an endorsement through the North Carolina Infant and Early Childhood Mental Health Association by 2030 (D. Kennerson, T. Van Newkirk, A. Wolfe, B. Polanik, A. Bailey, J. Sayles & J. Drake-Croft, personal communication, 21 March 2024).

## 9. Conclusions

Collaboration between infant and early childhood mental health and early childhood intervention services is crucial for the overall well-being of young children. The additional advantage that each program offers to the other sets the stage for services that are more responsive to family priorities, needs, and concerns. When all stakeholders have a comprehensive understanding of a child’s needs and form a system of care, better outcomes can be reached with lifelong implications. Although much progress has been made in how mental health is incorporated into ECI systems over the last twenty years, IECMH is not a routine service. As our understanding of the importance of early childhood social-emotional development increases, it is critical that at every level mental health providers and ECI professionals collaborate to create a comprehensive system.

Additional research is needed to effectively assess the advantages of collaboration between Early Childhood Intervention (ECI) and Infant and Early Childhood Mental Health (IECMH). Research on the prevalence of IECMH intervention is critical to determine workforce needs. Determining how extensive ECI provider agencies are at referring to IECMH is needed to determine advocacy and awareness efforts. Assessing the knowledge and competency of ECI providers in implementing IECMH strategies such as the Pyramid model is important to determine professional development opportunities. Finally, determining if incorporating IECMH services into ECI increases the effectiveness of ECI services, especially meeting ECI outcome 1: the child has positive social-emotional skills.

In addition to research, a variety of recommended practices can enhance this collaboration:

1. Early Detection and Treatment: Ensure that all domains of development are assessed by all child-serving providers. ECI and IECMH provider teams should collaborate to ensure that all areas are being assessed appropriately, cross-provider training is being implemented to ensure competency and reflective supervision is done to reinforce competency and build confidence as needed;
2. Collaborate with Child Welfare: The ECI and IECMH teams should reach out to Child Welfare agencies to closely examine policies and practices related to the screening and referral of infants and toddlers who have experienced early life stressors;
3. Cultural Humility & Equity: Both fields emphasize culturally responsive practices. By combining efforts, they can foster a system of care characterized by inclusive approaches that respect diverse family structures, beliefs, and values. This ensures that services are relevant and accessible to all;
4. Resiliency Building: Integrated approaches that include principles of mental health in early intervention and vice versa can help build resilience in both the child and the family system. This is particularly important for families facing additional stressors or challenges, such as those facing a diagnosis or limited resources;
5. Parental Support and Education: Parents and caregivers receive education and support that address both the developmental and emotional needs of their children, enabling them to actively participate in interventions and reinforce positive changes at home;
6. Professional Development: Cross-discipline professional development to strengthen the knowledge base of ECI and IECMH providers. Enhancements might include universal training to ensure foundational skills in promoting positive parent–child interactions and identifying children with developmental concerns;
7. Advocacy and Policy Influence: Plan and implement new policies that strengthen the capacity of the ECI program and the IECMH program to collaborate across sectors;
8. Reduction of Service Fragmentation: Create policies and procedures that reduce the fragmentation and duplication of services, helping families to navigate the system and receive consistent and uninterrupted care.

In conclusion, integrating the expertise of infant and early childhood mental health professionals with the developmental focus of early intervention services fosters a comprehensive approach to care that significantly enhances outcomes for young children. Unified efforts ensure a seamless, well-rounded, and nurturing environment for the child’s growth and development across all life domains.
